# The evidence and the possible significance of autophagy in degeneration model of human cervical end-plate cartilage

**DOI:** 10.3892/etm.2013.1465

**Published:** 2013-12-30

**Authors:** HONGGUANG XU, SHOULIANG XIONG, HONG WANG, MIN ZHANG, YUNFEI YU

**Affiliations:** Department of Orthopedic Surgery, Yijishan Hospital, Wannan Medical College, Wuhu, Anhui 241001, P.R. China

**Keywords:** autophagy, endplate chondrocytes, intervertebral disc

## Abstract

The aim of this study was to observe autophagy in chondrocytes from degenerative human cervical vertebral end-plates and to investigate the significance of variations in autophagy in the degeneration of cervical vertebral end-plate chondrocytes. Cartilage end-plates were obtained from 48 inpatients admitted to hospital between February 2011 and August 2012. The patients were divided into the control group (n=17) with cervical vertebral fracture or dislocation and the cervical spondylosis group (n=31) with cervical spondylotic myelopathy. End-plate chondrocytes were isolated via enzyme digestion and then cultured *in vitro*. The cells were stained with toluidine blue and hematoxylin-eosin (H&E). A laser scanning confocal microscope and monodansylcadaverine (MDC) were used to reveal autophagy in the end-plate chondrocytes. Reverse transcription polymerase chain reaction (RT-PCR) was used to detect mRNA expression of type II collagen and aggrecan. Western blotting was conducted to detect LC3 proteins. The chondrocytes isolated from the degenerative human cervical end-plates were cultured successfully *in vitro*. The morphology of the cells from the cervical spondylosis group tended to exhibit changes in spindle morphology compared with the control group. Autophagic bodies were stained with MDC. LC3 proteins were visible in the intracellular and perinuclear regions under the laser scanning confocal microscope. The mRNA expression levels (relative to those of β-actin) of aggrecan (0.715±0.194) and type II collagen (0.628±0.254) in the cervical spondylosis group were markedly decreased compared with those in the control group (0.913±0.254 and 0.845±0.186, respectively; both P<0.05). The LC3-II/LC3-I ratio was observed to be significantly reduced in the cervical spondylosis group by Western blot analysis. Autophagy has an important role in human cervical disc degeneration. The regulation of autophagy may prevent disc degeneration in cartilage end-plate cells.

## Introduction

The aging population in China is becoming increasingly evident along with the acceleration in the pace of life. The number of individuals with neck pain has also increased, but the pathogenesis of this health problem remains unclear. The cartilage end-plate is an important part of the intervertebral disc. The degeneration of this end-plate is closely associated with intervertebral disc degeneration, which is a cell-mediated process. The chondrocytes are the major cell type in the cartilage end-plate. These cells have an important role in maintaining the physiological functions of the intervertebral disc and the integrity of the extracellular matrix (1). Apoptosis is considered an important factor in disc degeneration (2). Extensive clinical and animal model studies have shown that cell structure loss and cell death are associated with intervertebral disc degeneration (3). Therefore, studying the pathological physiology of chondrocytes is important.

Autophagy is a form of apoptosis. In this process, cells engulf cytoplasmic proteins or organelles which are then packed into vesicles, and form an autophagolysosome with the lysosome. This process modulates metabolic materials and certain organelles. The autophagic process has four parts: substrate-induced porautophagosome, autophagy, fusion of the autophagosome with lysosomes and degradation of the contents of the autophagosome (4). Autophagy has a significant role in various degenerative pathological processes. For example, this mechanism is associated with cancer, microbial infections, heart diseases and even life extension (5,6). However, the association between autophagy and cartilage end-plate degeneration has rarely been studied.

In this study, surgically removed cartilage end-plates from patients with cervical spondylosis were used to establish degenerative chondrocyte cultures through enzyme digestion. Cultured chondrocytes obtained from the cartilage end-plates of patients with cervical vertebral fracture or dislocation served as the control. In addition, autophagy in the cultured chondrocytes was observed and the significance of variations in autophagy in the degeneration of cervical vertebral end-plate chondrocytes was investigated.

## Materials and methods

### Subjects

**Figure 1:**
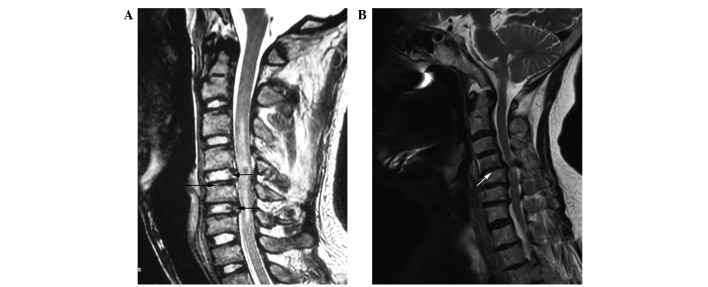
Representative MRI of patients. (A) Control patient; the black arrows indicate the experimental material positions. The C_4_, C_5_ cervical vertebrae are fractured and dislocated, whereas the disc signals of C_4–5_, C_5–6_ are normal. (B) Cervical spondylosis group; white arrows indicate the surgical subtotal vertebrectomy. The discs of C_4–5_, C_5–6_ and C_6–7_ are herniated; the signals indicate clear disc degeneration in the cervical spondylosis group.

The subjects were cervical spine surgery patients admitted to Yijishan Hospital, Wannan Medical College (Wuhu, China) between February 2011 and August 2012. A total of 31 cases of cervical spondylosis (the cervical spondylosis group) were selected, with 19 males and 12 females, aged 38 to 72 years. The average age was 52 years. Seventeen cases of fracture and dislocation patients (the control group) were also selected, with 11 males and 6 females, aged 23 to 36 years. The average age was 30 years. Patients with tumors, tuberculosis, diabetes, infections and metabolic bone diseases were excluded. All the patients underwent magnetic resonance imaging (MRI) examination prior to surgery. The degeneration of pathological grade cartilage of the end-plate and intervertebral disc was classified according to Miller (7) and Thompson *et al* (8). Representative MRI results are shown in Fig. 1. The control group had 5 cases of Miller grade 0 and 12 cases of Miller grade 1 according to MRI examination of the cartilage end-plate prior to surgery. After surgery, 7 patients did not exhibit pathological degeneration. A total of 10 cases of pathological disc degeneration were Thompson grade 1. The cervical spondylosis group had 8 cases of Miller grade 2 and 23 cases of Miller grade 3 according to MRI examination of the cartilage end-plate prior to surgery. In addition, 6 cases were Thompson grade 3, 15 cases were Thompson grade 4 and 10 cases were Thompson grade 5 according to MRI examination of disc degeneration. This study was conducted in accordance with the Declaration of Helsinki and with approval from the Ethics Committee of Wannan Medical College. Written informed consent was obtained from all participants.

### Chondrocyte isolation and culture

The cartilage end-plate tissue samples of the patients were excised from the disc and immediately sent to the central laboratory (Yijishan Hospital, Wannan Medical College, Wuhu, China). The samples were placed under a dissecting microscope (magnification, ×4) and processed by aseptic technique in a biological safety cabinet. The cartilage end-plate, nucleus pulposus and annulus of the samples were separated carefully. The chondrocytes were isolated (Type II collagenase and trypsin; HyClone, Logan, UT, USA) and cultured as in preliminary studies (9). The seeding density and culture conditions of the two groups of cells were the same. Changes in morphology and growth were observed regularly under an inverted microscope (Olympus, Tokyo, Japan).

### Toluidine blue and hematoxylin-eosin (H&E) staining

The two groups of cells were mounted on a coverslip. On day 9 of cell culture, ~60% fusion was observed. The cells were then removed, seeded and stained. i) Toluidine blue staining: the coverslip was washed thrice with phosphate buffer and fixed with 4% paraformaldehyde for 30 min. The coverslip was washed with water for 15 min and stained with toluidine blue for 4 h. After the coverslip was washed with tap water, the cells were air-dried, neutral gum was added and the cells were observed under a microscope (Olympus, Japan). ii) H&E staining: the coverslip was washed thrice with phosphate buffer and fixed with 4% paraformaldehyde for 30 min. The coverslip was washed with water for 15 min and stained with H&E. After the coverslip was washed with tap water, the cells were air-dried, neutral gum was added and the cells were observed under a microscope.

### Monodansylcadaverine (MDC) fluorescence staining

The two groups of cells were passaged and cultured. When the logarithmic growth phase of the cells reached a density of 70–80%, the digested cells were mixed and blown into a single-cell suspension. Cells (10 μl) were collected and counted at 5×10^4^/ml cells per well, with an additional 2 ml for each well. The cells were cultured for 24 h after adhesion and washed twice for 5 min with phosphate-buffered saline (PBS). The cells were treated with MDC (0.05 mmol/l; Sigma, St. Louis, MO, USA) diluted with PBS at 37°C for 15 min and then washed with PBS. The cells were observed under a fluorescence microscope (Leica, Wetzlar, Germany) with an emission filter of 356 and 545 nm.

### Laser scanning confocal microscopy

Logarithmic phase cells were trypsinized and the precipitate was collected following centrifugation. The cells were drawn up using a pipette and mixed with a RPMI-1640 medium without antibiotics, producing a final count of 2×10^5^ cells per well. Immunofluorescence was obtained following cell adhesion for 24 h. The specific steps were as follows. Fixing: The cells were washed thrice with PBS for 5 min each time, then fixed with 4% paraformaldehyde for 15 min. Blocking: The cells were washed thrice with PBS for 5 min each time. Fetal bovine serum albumin (5%) was added to the blocking solution at 37°C for 2 h. The cells were washed thrice with PBS for 5 min each time, anti-LC3B antibody (5 μg/ml, Sigma) was added at 4°C and the cells were incubated overnight. The cells were then washed thrice with PBS for 5 min each time. The diluted fluorescent secondary antibody [Alexa Fluor^®^ 555 donkey anti-rabbit IgG (H+L); 10 μg/ml; Santa Cruz Biotechnology, Inc., Santa Cruz, CA, USA] was added to the cells at 37°C for 1.5 h. The cells were washed thrice with PBS for 5 min each time. MDC (50 μM) was added to the cells as a dye at 4°C for 30 min and kept in the dark. A confocal laser scanner (Molecular Devices, Silicon Valley, CA, USA) was used to observe and capture photos of the cells the following day.

### Reverse transcription polymerase chain reaction (RT-PCR)

One-step TRIzol (Invitrogen, Carlsbad, CA, USA) was used to extract total RNA from cells. The purity and concentration were measured using an ultraviolet spectrophotometer (Olympus, Japan). A total of 3 μg RNA was extracted to synthesize cDNA. Aggrecan, type II collagen and β-actin were amplified using 3 μl cDNA as a template. β-actin was used as the internal reference. The gene primer sequences and the amplified fragment sizes are shown in Table I. The reaction conditions were as follows: predenaturation at 94°C for 5 min, denaturation at 94°C for 1 min and annealing at 56.1°C for 30 sec. Type II collagen was maintained at 60°C for 30 sec. β-actin was maintained at 51.9°C for 30 sec, extended at 72°C for 40 sec and amplified for 35 cycles. The reaction was terminated at 72°C after 5 min. The products were stored at 4°C. The PCR products were separated through 1.5% agarose gel electrophoresis. The Tanon Gel Image system 1D (Tanon, Shanghai, China) was used for semi-quantitative analysis. The relative band intensity percentages of the target gene and its internal reference were considered as the relative expression levels of the target gene mRNA for statistical analysis.

### Western blotting

Radioimmunoprecipitation assay buffer and phenylmethanesulfonyl fluoride were used to extract protein from the two groups of cells. The total protein was measured using the bicinchoninic acid method. Sodium dodecyl sulfate-polyacrylamide gel electrophoresis was performed on the protein. Human anti-LC3B, and anti-β-actin were diluted (1:1,000) in TBS-Tween 20 containing 1% BSA. The membranes were incubated for 2 h at 37°C with the primary antibody, washed three times in TBS-Tween 20 and for 1 h with the secondary antibody goat anti-rabbit IgG-HRP and goat anti-mouse IgG-HRP (1:5,000). Subsequent detection was performed using the ECL western blotting system (Amersham Biosciences, Chalfont St Giles, UK) according to the manufacturer’s instructions. Specificity of the antibody was assessed by omitting the first antibody in western blotting experiments.

### Statistical analysis

Experimental data are expressed as mean ± SD. The two groups were compared using two independent sample Student’s t-tests. The results were determined using SPSS 18.0 software (SPSS, Inc., Chicago, IL, USA). P<0.05 was considered to indicate a statistically significant result.

## Results

### Morphological changes of the chondrocytes

**Figure 2:**
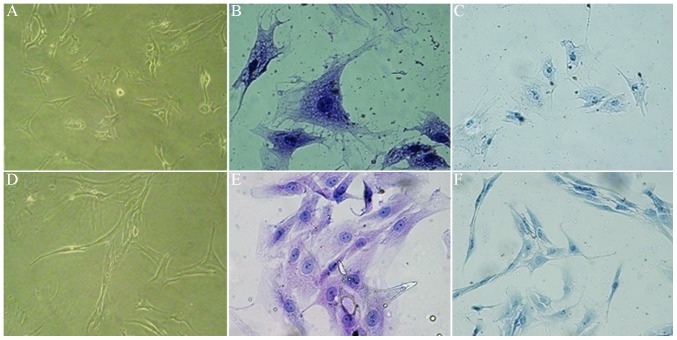
Morphological changes of the chondrocytes. (A–C) Control group. (D–F) Cervical spondylosis group. (A and D) The morphology of the two groups of chondrocytes cultured for 9 days (magnification, ×40). (B and E) Control group and cervical spondylosis group stained with H&E (magnification, ×100). (C and F) Control group and cervical spondylosis group stained with toluidine blue (magnification, ×100). H&E, hematoxylin-eosin.

Under an inverted microscope, it was observed that the majority of the control cells were polygonal. The cells from the patients with cervical spondylosis gradually stretched and the majority of these cells were shuttle type. The growth rate of the cells in the cervical spondylosis group was significantly slower than that of the cells in the control group. H&E staining demonstrated that the cytoplasms contained particulate matter in the control group; the nuclei were large and round, and two to three nucleoli per cell were observed. The spindle was the primary morphology of the cell nucleus in the cervical spondylosis group. The nucleolus was unclear and the gap between cells increased. The cytoplasm and cells surrounded by chondrocytes were stained and metachromatic in the two groups (Fig. 2). This result confirmed that the cells used in this experiment were end-plate chondrocytes.

### MDC fluorescence staining

**Figure 3:**
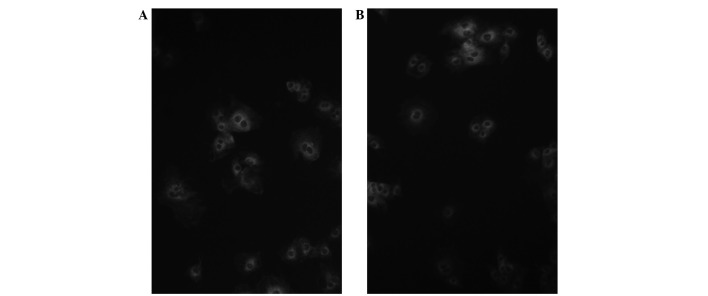
Autophagosomes stained with monodansylcadaverine (MDC) in chondrocytes (magnification, ×200). (A) Control group. (B) Cervical spondylosis group.

Under a dark field fluorescence microscope, the autophagosome absorbed MDC and appeared as a green granular structure. The control and cervical spondylosis groups both contained fluorescent granular autophagosomes (Fig. 3).

### Laser scanning confocal microscopy

**Figure 4:**
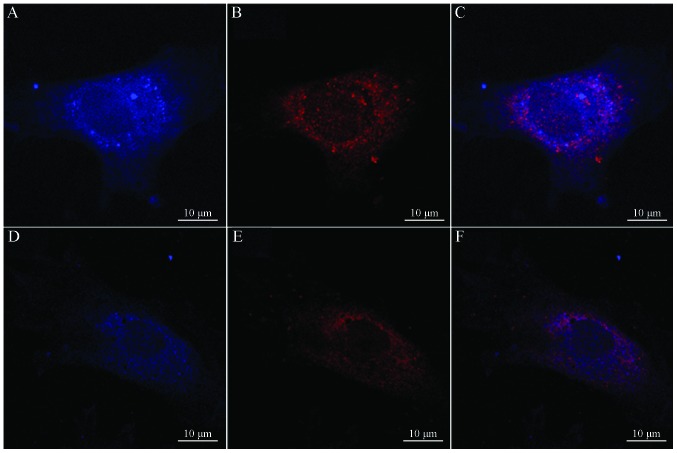
Images of confocal microscopy. (A and D) Blue indicated LC3 protein in the cell. (B and E) Red indicated the autophagosome position in the cell. (C) Superimposed images of (A) and (B). (F) Superimposed images of (D) and (E). (A–C, control group; D–F, cervical spondylosis group).

Laser scanning confocal microscopy showed that the LC3 proteins of the autophagosome were primarily concentrated in the intracellular and perinuclear regions (Fig. 4).

### RT-PCR

**Figure 5:**
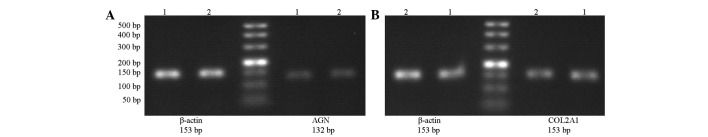
Electrophoresis patterns of (A) aggrecan and (B) type II collagen mRNA expression. Lane 1, control group; lane 2, cervical spondylosis group.

The mRNA expression levels of the aggrecan gene (0.715±0.194) and the type II collagen gene (0.628±0.254) in the cervical spondylosis group were much lower than those in the control group (0.913±0.254 and 0.845±0.186, respectively). The difference between the two groups was statistically significant (both P<0.05; Fig. 5).

### Western blotting

**Figure 6:**
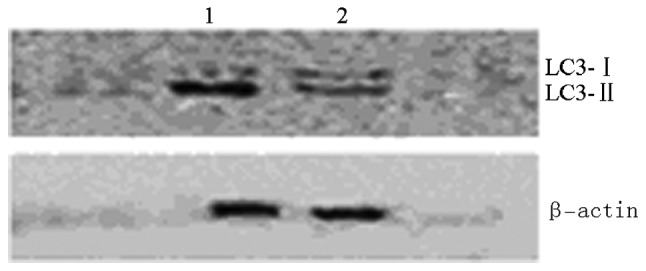
Expression of LC3-I and LC3-II in the control and cervical spondylosis groups detected by Western blotting. Lane 1, control group; lane 2, cervical spondylosis group.

The expression of LC3-II in the cervical spondylosis group was downregulated compared with that in the control group. LC3-I was upregulated, whereas the ratio LC3-II/LC3-I was also decreased. The expression of β-actin did not differ between the two groups (Fig. 6).

## Discussion

The cartilage end-plate constitutes the upper and lower bounds of the intervertebral disc. This disc is the largest avascular structure in the body with a relative lack of nutrient supply. The supply of nutrients and the discharge of metabolites in the disc are conducted through the end-plate. Therefore, the cartilage end-plate has an important role in the nutrition path of the disc, since material exchange occurs between the disc and its external environment. The delivery of nutrients to the cartilage end-plate is primarily affected by the shape, size and charge of the solute, as well as the composition of the cartilage end-plate. The cartilage end-plate is composed of cells and stromata. The major cell types in the cartilage end-plate are chondrocytes and fibroblasts. The matrices are primarily composed of aggrecan and type II collagen. The maintenance of normal decomposition and anabolism by a matrix is essential for its biological function. The cartilage end-plate prevents small molecules called proteoglycans from being lost in the disc. Following the degeneration of the cartilage end-plate, this protective effect is weakened, thus the loss of disc proteoglycan increases (10–12). As the nutrition path of the cartilage end-plate breaks, the matrix syntheses of type II collagen and aggrecan, as well as the amount of water in the cartilage end-plate, are reduced. Consequently, disorders of the cartilage end-plate structure result and the normal function of the cartilage end-plate is lost, eventually leading to disc degeneration (13). In the present study, chondrocyte phenotypes were distinguished by toluidine blue staining, H&E staining and RT-PCR. The results show that the expression levels of type II collagen and aggrecan in the cervical spondylosis group end-plates were significantly lower than those in the control group. Furthermore, the results are in accordance with previous studies (9,14).

Autophagic cell death has been observed from as early as the 1960s; however, cause for concern has not been identified until recently. As an innate host defense mechanism, autophagy is involved in the removal of damaged organelles (15). This mechanism is an important physiological process for maintaining cellular homeostasis, such as in the growth and development of an organism, cell differentiation and proliferation, remodeling, and degradation of aging or damaged intracellular organelles, hamartoma and excess proteins (16). Autophagy is primarily involved in clearing damaged organelles and in reusing macromolecular substances of cells. Autophagy also has an important role in maintaining cell homeostasis and in promoting cell survival. In addition, autophagy serves as the main degradation pathway for macromolecular proteins and organelles. Autophagy has a protective effect on various tissue cells (17). Lapatinib induces autophagy and is able to impede the growth of breast cancer (18). The regulation of autophagy also prevents diabetes, which induces damage to the endoplasmic reticulum (19). Autophagy is closely associated with human osteoarthritis and the arthritis model in mice, and may have a protective effect against osteoarthritis (20). In addition, autophagy has an extremely important role in preventing damage to the mitochondrion and the endoplasmic reticulum, and may effectively remove damaged organelles, thus delaying or preventing apoptosis initiation, reversing cell injury or degeneration, or even death (21). If autophagy is enhanced in the degeneration of cartilage cells, then various metabolic waste materials in cartilage cells are promptly removed, thus allowing the cells to develop a suitable environment. The degeneration of articular cartilage may be delayed effectively or even blocked. Apoptosis has an important role in disc degeneration and numerous other diseases. Autophagy has a significant role in blocking apoptosis, however, the specific mechanisms of autophagy in disc degeneration remain unclear.

Autofluorescent MDC labels the autophagic structures of cells and may be used as an autophagy tracer (11). In the present study, autophagic bodies in the control and cervical spondylosis groups were observed through MDC staining. LC3, which is the human homolog of the autophagy-related gene Atg8 in mammalian cells, is also commonly used in detecting autophagy (22). The LC3 protein rapidly moves to an autophagic environment (23). In the present study, the LC3 proteins of the autophagosome were visible in the cytoplasm and in the perinuclear space under a laser scanning confocal microscope. These proteins appeared as dot-like structures, thus confirming that autophagy occurs in the chondrocytes of the intervertebral disc. Autophagy occurs in the degenerative discs of rats (24), as well as in human cervical vertebrae, as shown in the current study. LC3 has two forms: LC3-I and LC3-II. The latter is located in the membrane of the autophagosome and is considered as a marker of autophagy. As autophagy increases, LC3-II is upregulated and LC3-I is downregulated, thus the LC3-II/LC3-I ratio is proportional to autophagy (25). Western blotting results showed that compared with the control group, LC3-I in the cervical spondylosis group was upregulated, whereas LC3-II was downregulated and the LC3-II/LC3-I ratio decreased. The results are consistent with a previous study (26), and imply that autophagy has a negative correlation with the degeneration of chondrocytes.

Autophagy occurs extensively in the degradation and recirculation of system eukaryotic cells. This mechanism is an important physiological process that allows primary lysosomes to process endogenous substrates, and organisms to maintain the metabolic balance of proteins and the stability of the intracellular environment. In the present study, it was observed that autophagy has an important role in human cervical disc degeneration. The regulation of autophagy may prevent disc degeneration in cartilage end-plate cells. Information concerning autophagy has been increasing as numerous studies are conducted. However, few data on autophagy in chondrocytes, as well as its effect on the growth and degeneration of such cells, are available. In the present study, autophagy was demonstrated to be closely associated with the degeneration of chondrocytes at the molecular level. However, the specific role of autophagy in disc degeneration requires further study. The regulation activity of autophagy in chondrocytes may provide a new direction for the treatment of spinal degenerative diseases.

## Figures and Tables

**Table I:** Gene primer sequences and the amplified fragment lengths.

Gene	Forward primer	Reverse primer	Product length (bp)
Type II collagen	5′-CTCCTGGAGCATCTGGAGAC-3′	5′-ACCACGATCACCCTTGATCT-3′	153
Aggrecan	5′-AGTATCATCAGTCCCAGAATCTAGCA-3′	5′-AATGCAGAGGTGGTTTCACTCA-3′	132
β-Actin	5′-GCATCCCCCAAAGTTCACAA-3′	5′-AGGACTGGGCCATTCTCCTT-3′	153
